# The Microphone as a Diagnosticator of the Entire Physical Organism

**Published:** 1885-05

**Authors:** Edward Joseph Eve

**Affiliations:** Augusta, Ga.


VOL. II. MAY, 1885. NO. 3.

### Original Communications.

### THE MICROPHONE AS A DIAGNOSTICATOR OF THE ENTIRE PHYSICAL ORGANISM.

BY EDWARD JOSEPH EVE, M.D., AUGUSTA, GA.

Having heard some one casually remark, some months ago, that on the microphone " the walk of a fly was like the tramp of an elephant," this expression, hyperbolical and exaggerated as it was, yet conveyed to my mind a suggestion which contained an idea. The thought struck me, that this principle of acoustic being established, why not apply it to explore the sounds of the entire physical organism, viz.: the sounds of the heart, of the lungs, of the intestines, of the fœtal circulation, intra-cranial sounds, muscular sounds, to differentiate aneurisms from tumors, to hear crepitation in obscure cases of fracture, in explorations of the bladder for stone, and many other sounds now under investigation by me.

If argument were necessary to establish the philosophy of its use, the eye furnishes a ready one. The analogy is apparent. If in scientific research that wonderfully contrived organism requires aid in the use of the microscope; if its powers can be supplemented thus by artificial means, thereby aiding us in the investigation of disease, is it unreasonable to suppose that the ear can be supplemented in a like ratio ?

The announcement of so startling a discovery will doubtless engender antagonism and meet with unbelief in many quarters. This, of course, is expected. No discovery of any value within the scope of scientific research has escaped them.

I think I now hear an objection offered to its use, the one which will most primarily and naturally present itself. "Even admitting that the results claimed will follow, the sounds turned loose will be so multitudinous, tumultuous and chaotic that the diagnostician will be confused all the more."

If this reasoning proves anything it proves too much.

Shall we reject the useful because environed by that which cannot be utilized ? Shall not the wheat be garnered from the chaff ? Shall the few not be saved because of the many that are lost ? Shall that new and precious alkaloid, cocaine, that blessed anæsthetic, be rejected because of the dross which environs it ? Shall quinia, shall caffein, shall the eye be forever closed because some trivial objects meet its gaze ?

Shall the ear be forever stopped because some useless sounds vibrate upon its chord ? Let us accept the maxim, "*Je prends le bien ou je le trouve.*"

The objection offered against the utility of this *electric stethoscope*, on account of the multiplicity of sounds it evokes, is too sweeping in its denunciation. It admits of no discrimination. No one, after seriously studying the subject, will make so unphilosophical a statement. By referring to the examples of sound given in the beginning of this article, it will be seen that it cannot possibly apply to many of them, and to the few in a partial degree only. It is in the thoracic region that the greatest difficulties will have to be overcome. In using this instrument, we must first familiarize ourselves with the sounds of an organ, the heart, for instance, in a normal state, and then apply it in like manner to one in a diseased condition. Thus, by a judicious use of the instrument, founded upon experience, we learn to eliminate the superfluous sounds from such as assist in the diagnosis.

The skilled physician may hear many sounds, many vibrations may strike upon the tympanum of his ear, that will not be transmitted sensibly to the brain, and his attention will not be distracted by them. The eye, in looking intently upon a single object, sees many of which it takes no note.

But while I earnestly advocate the use of the microphone as above presented, and believe that its success is assured, I am not unmindful of the many difficulties which will be encountered in adapting this scientific principle to a practical use. Many formidable obstacles will obstruct the pathway to its new field of usefulness, which will require time, patience and skill to surmount. We have an example of this in the stethoscope. From what a rude beginning did it rise to its present state of efficiency and exactness. First, the *stethoscope of Laennec*, discovered in 1816—a quire of paper rolled into a cylinder. Then follow a long succession of instruments of various materials, all founded upon the same principle, for the conduction of sound, culminating in that most excellent and ingenious instrument, the *stethoscope of Camman*.

I now propose the introduction of a principle never before used in stethoscopy, viz.: *Electricity*.

I simply throw this out as a crude suggestion, with the sincere hope, that while I am engaged in devising means whereby this idea may assume a practical shape, some other of our profession, more skilled than myself in mechanics, and with better opportunities for the work, may develop the application of this truth.

And I would further suggest that an *audiphone*, constructed on this basis, might, I think, prove of inestimable value to those whose hearing is somewhat impaired.

Thus, we see the microphone, an instrument of comparatively little significance, as now used, suddenly found capable of being converted into one whose application is the most wonderful and useful in the whole realm of acoustics.

I have thus hastily presented a few only of the many points of interest which environ a subject of so great magnitude. May it prove a help to the physician and a blessing to the sick.

